# Unraveling templated-regulated distribution of isolated SiO_4_ tetrahedra in silicoaluminophosphate zeolites with high-throughput computations

**DOI:** 10.1093/nsr/nwac094

**Published:** 2022-05-13

**Authors:** Yan Li, Chao Shi, Lin Li, Guoju Yang, Junyan Li, Jun Xu, Qinfen Gu, Xingxing Wang, Ji Han, Tianjun Zhang, Yi Li, Jihong Yu

**Affiliations:** State Key Laboratory of Inorganic Synthesis and Preparative Chemistry, Jilin University, Changchun 130012, China; School of Textile and Clothing, Yancheng Institute of Technology, Yancheng 224000, China; State Key Laboratory of Inorganic Synthesis and Preparative Chemistry, Jilin University, Changchun 130012, China; State Key Laboratory of Inorganic Synthesis and Preparative Chemistry, Jilin University, Changchun 130012, China; State Key Laboratory of Inorganic Synthesis and Preparative Chemistry, Jilin University, Changchun 130012, China; State Key Laboratory of Magnetic Resonance and Atomic and Molecular Physics, National Center for Magnetic Resonance in Wuhan, Wuhan Institute of Physics and Mathematics, Innovation Academy for Precision Measurement Science and Technology, Chinese Academy of Sciences, Wuhan 430071, China; Australian Synchrotron, Clayton 3168, Australia; State Key Laboratory of Inorganic Synthesis and Preparative Chemistry, Jilin University, Changchun 130012, China; State Key Laboratory of Magnetic Resonance and Atomic and Molecular Physics, National Center for Magnetic Resonance in Wuhan, Wuhan Institute of Physics and Mathematics, Innovation Academy for Precision Measurement Science and Technology, Chinese Academy of Sciences, Wuhan 430071, China; State Key Laboratory of Inorganic Synthesis and Preparative Chemistry, Jilin University, Changchun 130012, China; State Key Laboratory of Inorganic Synthesis and Preparative Chemistry, Jilin University, Changchun 130012, China; State Key Laboratory of Inorganic Synthesis and Preparative Chemistry, Jilin University, Changchun 130012, China; International Center of Future Science, Jilin University, Changchun 130012, China; State Key Laboratory of Inorganic Synthesis and Preparative Chemistry, Jilin University, Changchun 130012, China; International Center of Future Science, Jilin University, Changchun 130012, China

**Keywords:** acidity, computational chemistry, heterogeneous catalysis, structure–activity relationships, zeolites

## Abstract

Silicoaluminophosphate (SAPO) zeolites are well-known catalytic materials because of the mild acidity originating from the isolated SiO_4_ tetrahedra in their frameworks. Regulating the distribution of isolated SiO_4_ tetrahedra in SAPO zeolites is formidably challenging because SiO_4_ tetrahedra tend to agglomerate to form Si islands and the isolated SiO_4_ tetrahedra are difficult to determine using conventional characterization techniques. Here we synthesized Si-island-free SAPO-35 zeolites by using *N*-methylpiperidine as a new template, which exhibited excellent thermal stability compared to conventional SAPO-35 zeolites and a substantially improved methanol-to-olefins catalytic lifetime even comparable to that of commercial SAPO-34 zeolites. More strikingly, with the aid of high-throughput computations on 44 697 structure models combined with various state-of-the-art characterization techniques, for the first time, we reveal that the host–guest interactions between template molecules and SAPO frameworks determine the specific distributions of isolated SiO_4_ tetrahedra, which are responsible for the improvement in the chemical properties of zeolites. Our work provides an insight into the template-based regulation of isolated SiO_4_ tetrahedra in SAPO zeolites, which opens a new avenue in the discovery of promising zeolite catalysts with optimal SiO_4_ distribution.

## Teaser

The template-regulated distribution of the isolated SiO_4_ tetrahedra in silicoaluminophosphate zeolite SAPO-35 was unraveled by high-throughput computations combined with various state-of-the-art characterization techniques.

## INTRODUCTION

Zeolites are among the most important heterogeneous catalysts in the chemical industry because of their shape selectivities and tunable active sites within their framework structures [1,2]. The active sites in zeolites can be generated around specific TO_4_ (T=Al, Si, P, etc.) tetrahedra in zeolite frameworks [3–5]. For example, the AlO_4_ tetrahedra in aluminosilicate zeolites provide negative charges to the zeolite frameworks, which afford Brønsted acidity favoring important catalytic reactions. The distribution of AlO_4_ tetrahedra determines the locations of active sites and the chemical properties of aluminosilicate zeolites [6–8]. Similarly, in silicoaluminophosphate (SAPO) zeolites, the SiO_4_ tetrahedra are responsible for the Brønsted acidity [9], as well as their catalytic performance, thermal stability and adsorption properties [10–13]. For instance, SAPO-34 zeolites are currently the most important commercial catalysts for methanol-to-olefins (MTO) conversion, which is an important reaction for the production of light olefins from non-petroleum resources [13,14].

Regulating the concentration and the distribution of active TO_4_ tetrahedra in zeolite frameworks plays an important role in the development of high-performance zeolite catalysts. For aluminosilicate zeolites, the distribution of AlO_4_ tetrahedra can be regulated by several approaches, such as the introduction of framework heteroatoms, the usage of alcoholic additives and the employment of different organic amines as the template molecules [7,15–20]. In particular, the design of template molecules has facilitated the formation of a wide range of aluminosilicate zeolites, in which the preferential T sites for AlO_4_ tetrahedra or the relative distances between AlO_4_ tetrahedra can be regulated [16,18–20]. For instance, Muraoka *et al.* revealed that different azoniabicyclo[2.2.2]octane molecules could alter the energetically favorable T sites for AlO_4_ tetrahedra in **IFR**-type zeolites [19], Di Iorio *et al.* utilized the cooperative and competitive structure-directing effect of organic ammonium cations and sodium ions to regulate the arrangement of isolated and paired AlO_4_ tetrahedra in chabazite **(CHA)** zeolites [20].

Compared to AlO_4_ tetrahedra in aluminosilicate zeolites, the distribution of SiO_4_ tetrahedra in the SAPO zeolite framework is more difficult to control because SiO_4_ tetrahedra tend to agglomerate via Si–O–Si linkages to form Si islands with reduced Brønsted acidity [9]. Thus, regulating the Si islands to tune the acidity and catalytic activity of SAPO zeolites has attracted much research attention [21–24]. However, suppressing the formation of Si islands and regulating the distribution of isolated SiO_4_ tetrahedra in SAPO zeolite frameworks is formidably challenging. This is because a large amount of Si in the synthetic system tends to form Si islands in SAPO zeolites, whereas a small amount of Si may slow the growth of SAPO zeolites and lead to a low product yield or the formation of impurities [25,26]. Meanwhile, the isolated SiO_4_ tetrahedra are usually distributed among all possible T sites and their exact locations cannot be unambiguously determined via conventional X-ray diffraction techniques [27]. As a result, the effects of the distribution of isolated SiO_4_ tetrahedra on the chemical properties of SAPO zeolites have not yet been identified so far.

Recently, high-throughput computations have demonstrated their power in the design of zeolite structures and template molecules, providing valuable guidance for the synthesis and property regulation of zeolites [28–31]. Here, for the first time, we report the template-regulated distribution of isolated SiO_4_ tetrahedra in a SAPO zeolite unraveled by high-throughput computations combined with various state-of-the-art characterization techniques. Using a new template molecule, *N*-methylpiperidine (NMP), we have synthesized SAPO-35 zeolites with **LEV**-type framework topology constructed by *lev* cages, single 6-rings and double 6-rings [32] (Supplementary Fig. S1). Compared to the SAPO-35 zeolites prepared using the conventional templates, e.g. hexamethyleneimine (HMI) [12,33,34], those prepared using NMP showed excellent thermal stability when comparing samples of the same or similar chemical composition. Moreover, contrary to the well-accepted fact that conventional SAPO-35 zeolites are not good MTO catalysts because of their limited lifetime [33–35], SAPO-35 zeolites synthesized using NMP exhibit a substantially improved MTO lifetime. More importantly, with the aid of high-throughput computations based on 44 697 SAPO-35 structure models combined with solid-state ^29^Si MAS NMR and synchrotron X-ray diffraction (XRD), we are able to unravel the mysterious distributions of isolated SiO_4_ tetrahedra, which result from the different structure-directing effects of distinct template molecules and are responsible for the property improvement of SAPO zeolites. This work reveals that the distribution of isolated SiO_4_ tetrahedra in SAPO zeolites can be regulated by the host–guest interactions provided by template design, opening a new avenue to the rational tuning of the chemical properties of SAPO zeolites.

## RESULTS AND DISCUSSION

### Synthesis and characterization

Using a new template NMP and the conventional template HMI (Supplementary Fig. S2), we synthesized a series of SAPO-35 zeolites with different particle sizes via conventional hydrothermal synthesis as well as seed-assisted microwave irradiation (Supplementary Table S1). Details about the synthesis and the characterizations of these samples are provided in the supporting information. All these samples are denoted as SAPO-35_*x*_*y*_*z*, where *x* could be ‘NMP’ or ‘HMI’, representing the template used for the synthesis; *y* could be ‘m’ or ‘n’, which represents either a microscale or a nanoscale particle size; *z* represents the Si content in SAPO-35 zeolites calculated as Si/(Si + P + Al) in molar ratio. In general, SAPO-35 zeolites synthesized with NMP exhibited a wide range of Si contents from 5% to 23%. In particular, the Si content in SAPO-35_NMP zeolites could be as low as 5%, which is among the lowest Si contents for all known SAPO zeolites [23,25,36–38], thus favoring the formation of isolated SiO_4_ tetrahedra. In comparison, the lowest Si content in SAPO-35 zeolites prepared using conventional HMI was 8%. Further decreasing the Si content in SAPO-35_HMI led to impurities. Notably, the synthesis of SAPO-35 using NMP as the template was generally easier to reproduce than using HMI. All these results indicate that NMP is a more suitable template to regulate the Si content in SAPO-35 zeolites than conventional HMI.

As shown in Supplementary Figs S3 and S4, the as-prepared SAPO-35 samples are all uniform rhombohedral crystals with different particle sizes. The use of seed-assisted microwave irradiation greatly reduced the crystal sizes. In particular, SAPO-35_NMP_n_0.05 exhibits a crystal size as small as 170 nm, which is ∼100-fold smaller than SAPO-35 samples synthesized under conventional hydrothermal conditions. Supplementary Fig. S5 shows the XRD patterns of all of the as-synthesized SAPO-35 samples, which are in good agreement with the characteristic pattern of **LEV**-type zeolites [32]. All of the samples are highly crystalline and no impurity is observed.

The N_2_ adsorption–desorption isotherms of the calcined SAPO-35 samples are shown in Supplementary Fig. S6, which exhibit characteristic type I isotherms of microporous zeolites. All of the SAPO-35 samples show high surface areas (451–538 m^2^ g^–1^) and possess similar micropore volumes in the range of 0.19–0.24 cm^3^ g^–1^, which are similar to previous SAPO-35 reported in literature [39].

### Thermal stability and catalytic performance

**Figure 1.**
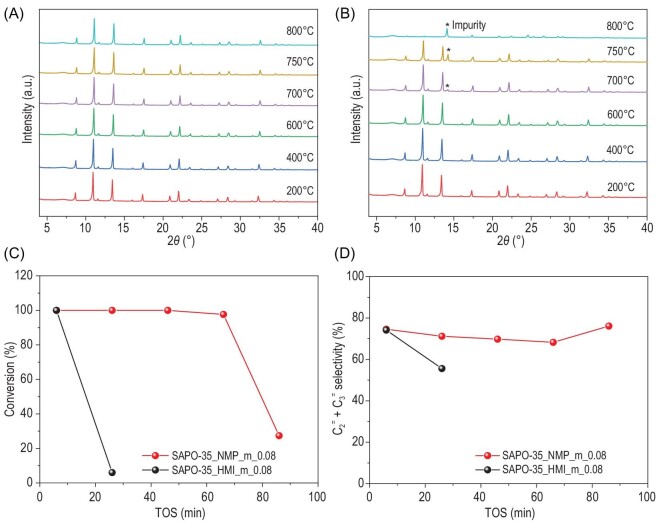
Variable temperature X-ray diffraction patterns of (A) SAPO-35_NMP_m_0.08 and (B) SAPO-35_HMI_m_0.08. (C) Methanol conversion and (D) selectivity of ethylene and propylene varying with time-on-stream over SAPO-35_NMP_m_0.08 and SAPO-35_HMI_m_0.08 during MTO reaction, respectively. Experimental conditions: WHSV = 2 h^–1^, T = 400°C, catalyst weight = 200 mg.

To investigate the difference in the structures and properties of SAPO-35 prepared using NMP and HMI, we took SAPO-35_NMP_m_0.08 and SAPO-35_HMI_m_0.08 with similar Si contents and crystal sizes for further characterizations. Temperature-programmed desorption of ammonia (NH_3_-TPD) measurements indicate that these two samples also possess similar acidities (Supplementary Fig. S7). Despite above-mentioned similarity, these two samples exhibited different chemical properties. Figure 1A and B shows the XRD patterns of these two samples heated at different temperatures. SAPO-35_NMP_m_0.08 remained stable at 800°C and no impurity peak was observed upon calcination. However, when SAPO-35_HMI_m_0.08 was heated to 700°C, impurity peaks appeared in its XRD pattern. The occurrence of impurities was also confirmed by the inset SEM images of the heated samples (Supplementary Fig. S4). These results indicate that SAPO-35_NMP_m_0.08 is thermally more stable than SAPO-35_HMI_m_0.08.

The catalytic performance of SAPO-35_NMP_m_0.08 and SAPO-35_HMI_m_0.08 was evaluated using MTO reactions at 400°C in a fixed-bed reactor (see the supporting information for details). These two samples show similar selectivity to C_2__-3_^=^. However, SAPO-35_NMP_m_0.08 shows a significantly longer catalytic lifetime than SAPO-35_HMI_m_0.08 (Fig. 1C and D), although they possess almost identical Si contents, particle sizes and acidity. So we assume such differences might originate from the distinct distributions of SiO_4_ tetrahedra in the frameworks of these two zeolite samples.

### Distribution of SiO_4_ tetrahedra

**Figure 2.**
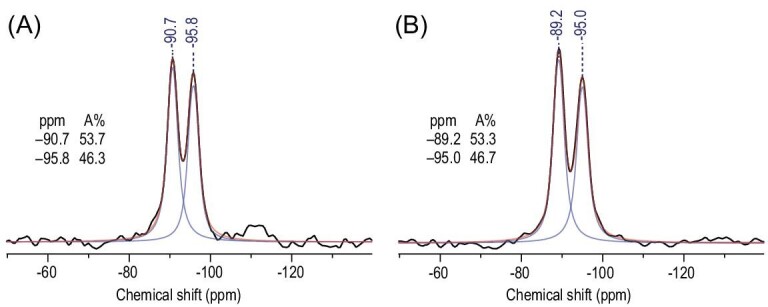
^29^Si MAS NMR of (A) SAPO-35_NMP_m_0.08 and (B) SAPO-35_HMI_m_0.08.

Both SAPO-35_NMP and SAPO-35_HMI possess the **LEV**-type zeolite framework, which consists of double 6-rings, single 6-rings and *lev* cages formed by these rings. There are two possible T sites for SiO_4_ tetrahedra to occupy, including the T1 site at the double 6-rings and the T2 site at the single 6-rings (Supplementary Fig. S1). To probe the preferential distribution of SiO_4_ tetrahedra, solid-state ^29^Si MAS NMR and synchrotron-radiation single-crystal XRD were performed on these two samples (Fig. 2 and Supplementary Table S2). The ^29^Si MAS NMR spectra for these two samples are almost identical, both showing only two peaks at –90 and –95 ppm. Thus, the two peaks at –90 and –95 ppm for both samples could be assigned to the isolated SiO_4_ tetrahedra at the T1 and T2 sites in the SAPO-35 framework, respectively [39]. However, the difference in the NMR patterns of these two SAPO-35 samples seems negligible. The absence of the peak of Si islands, which usually appear between –108 and –114 ppm, implies that Si atoms are incorporated into the SAPO-35 following the SM2 mechanism [9,39]. Synchrotron-radiation single-crystal XRD gives the cell parameters for SAPO-35_NMP as *a* = 13.176 Å and *c* = 45.914 Å, respectively, and those for SAPO-35_HMI as *a* = 13.264 Å, *c* = 45.355 Å, respectively. According to the SM2 mechanism, Si atoms will occupy P sites only, but the average P–O bond lengths do not show much difference between the T1 and T2 sites (1.530 and 1.528 Å for SAPO-35_NMP, and 1.532 and 1.536 Å for SAPO-35_HMI; details are provided in Supplementary Table S3, CIF-S1_AlPO-35.cif, CIF-S2_SAPO-35_NMP_m_0.08.cif, CIF-S3_SAPO-35_HMI_m_0.08.cif). These results indicate that there is no obvious preferential occupancy for Si atoms among the two T sites in both samples.

**Figure 3.**
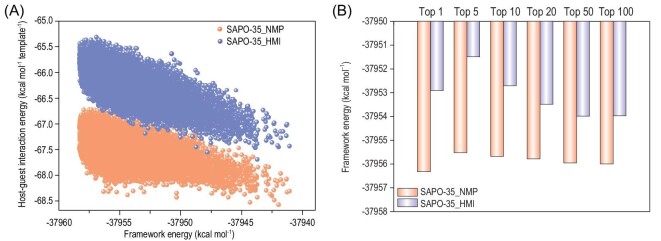
(A) Host–guest interaction energies between SAPO-35 frameworks and template molecules. (B) Framework energies of the top 100 stable host–guest models for each type of template molecule calculated.

Since all our available experimental characterization approaches could not identify the difference in the distribution of isolated SiO_4_ tetrahedra in SAPO-35_NMP and SAPO-35_HMI, we conducted high-throughput computations to reveal this mystery (see the supporting information for computational details). We built up an AlPO-35 structure model consisting of 54 Al sites and 54 P sites. To reduce the computational complexity, we introduced five isolated SiO_4_ tetrahedra in the unit cell of AlPO-35, which was closed to the lowest Si content (5%) achieved in this study, and enumerated all possible SAPO configurations for the distribution of five Si atoms among the 54 P sites following the SM2 mechanism. According to the NMR result that no Si islands were observed, the direct Si–O–Si linkage was avoided during our enumeration. Meanwhile, from NMR data, we could deduce that the numbers of Si atoms at T1 and T2 sites should be similar. So during structure enumeration, we only kept models with two Si atoms at T1 sites and three Si atoms at T2 sites, and models with three Si atoms at T1 sites and two Si atoms at T2 sites. In the end, a total of 44 697 distinct SAPO-35 structure models were built and all these models were geometrically optimized using the molecular mechanics method. NMP and HMI molecules were then put into these SAPO-35 models using molecular dynamics simulations, respectively. Finally, the host–guest models, i.e. SAPO-35 models with NMP or HMI molecules in their pores, were fully relaxed and the total energies of the host–guest models as well as the non-bonding host–guest interaction energies were calculated to evaluate the structure-directing effects of NMP and HMI. The relaxed host–guest models possessed cell parameters similar to the experimental ones measured using synchrotron-radiation single-crystal XRD, validating the feasibility of our high-throughput computations (Supplementary Table S2). In general, NMP shows a stronger structure-directing effect towards SAPO-35 than HMI (Fig. 3A and Supplementary Table S4), which agrees with the experimental fact that the synthesis of SAPO-35 using NMP was easier than using HMI. Moreover, we find that the most stable host–guest models induced by NMP exhibit significantly lower framework energies than the most stable host–guest models induced by HMI (Fig. 3B and Supplementary Table S4). This implies that NMP favors low-energy SAPO-35 frameworks, whereas HMI favors SAPO-35 frameworks with relatively high energies. This agrees with the experimental fact that SAPO-35 induced by NMP is thermally more stable than that induced by HMI. Since these high-throughput computational models successfully reproduce the difference in experimental lattice parameters and thermal stability, investigating the distribution of isolated SiO_4_ tetrahedra in these structure models might reveal the true difference between SAPO-35_NMP and SAPO-35_HMI.

**Figure 4.**
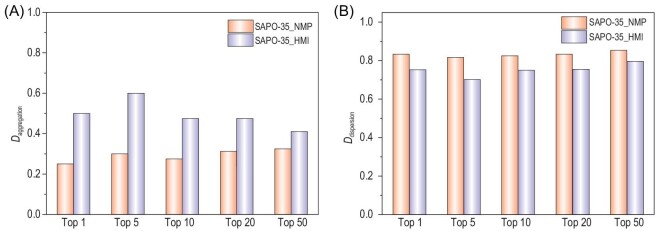
(A) Degree of aggregation (*D*_aggregation_) and (B) degree of dispersion (*D*_dispersion_) of isolated SiO_4_ tetrahedra in SAPO-35 zeolites.

To understand the distribution of isolated SiO_4_ tetrahedra in a quantitative way, we propose *D*_aggregation_ and *D*_dispersion_ to evaluate the degree of aggregation and the degree of dispersion of isolated SiO_4_ tetrahedra in our SAPO models, where *D*_aggregation_ is defined as the summation of the numbers of the neighboring SiO_4_ tetrahedra in adjacent 6-ring layers for each SiO_4_ tetrahedron divided by the upper limit of this summation (which is 8 for five Si atoms) and *D*_dispersion_ is defined as the number of *lev* cages containing Si atoms divided by 12, the total number of *lev* cages in each of our SAPO-35 models. A *D*_aggregation_ of 0.0 indicates that all of the SiO_4_ tetrahedra are highly dispersed and a *D*_aggregation_ of 1.0 indicated all of the Si atoms are aggregated and distributed among adjacent 6-ring layers. On the other hand, a *D*_dispersion_ of 1.0 indicates that the SiO_4_ tetrahedra are well dispersed among all *lev* cages, and a low *D*_dispersion_ indicates that the SiO_4_ tetrahedra are not well dispersed and distributed among a small number of *lev* cages. As shown in Fig. 4 and Supplementary Table S4, the most stable SAPO-35 zeolites induced by NMP possess much lower *D*_aggregation_ and higher *D*_dispersion_ than those induced by HMI, indicating that the isolated SiO_4_ tetrahedra are better dispersed in SAPO-35_NMP than SAPO-35_HMI.

**Figure 5.**
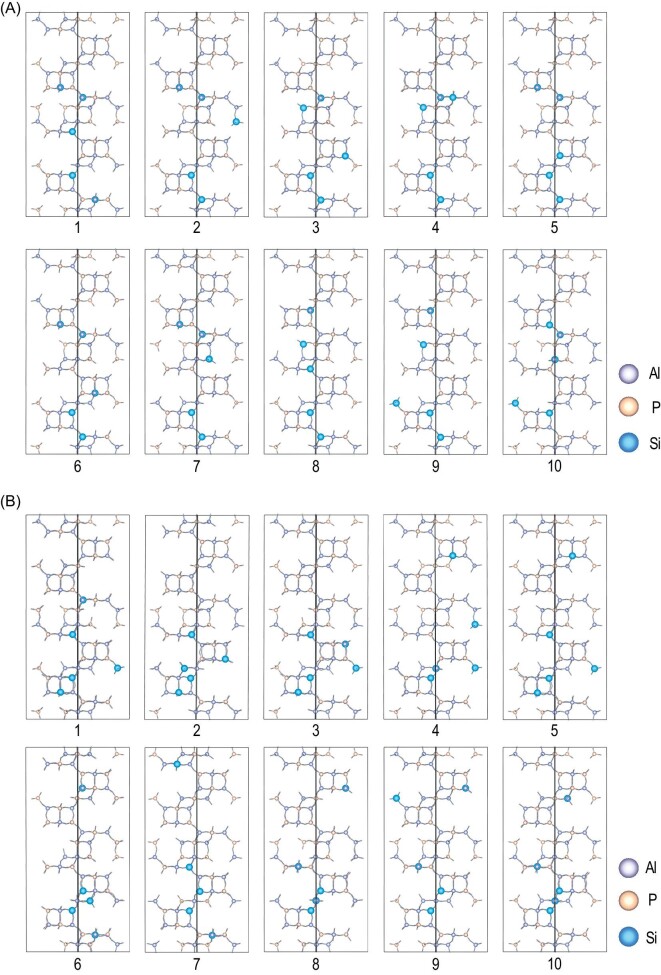
Top 10 stable host–guest models for (A) SAPO-35_NMP and (B) SAPO-35_HMI calculated via high-throughput computations. Template molecules were omitted for clarity.

The top 10 stable host–guest models for SAPO-35_NMP and SAPO-35_HMI calculated via high-throughput computations are shown in Fig. 5 and Supplementary Fig. S8. According to our computational results, the SAPO-35 zeolites induced by NMP possess more dispersed SiO_4_ tetrahedra distribution, which stabilizes zeolite frameworks because the negative charges introduced by isolated SiO_4_ are well dispersed. These results agree with our experimental observation that SAPO-35_NMP is thermally more stable than SAPO-35_HMI as well as the theoretical studies in the literature that maximizing the distances between SiO_4_ tetrahedra would be beneficial to the stability of SAPO frameworks [40,41]. Meanwhile, the dispersed distribution of SiO_4_ tetrahedra is generally favorable to MTO conversion, because the close contact of acid sites would induce side reactions that deactivated the MTO conversion [42,43]. This might be the reason why the SAPO-35 zeolites induced by NMP exhibit a longer catalytic lifetime than those induced by HMI.

Furthermore, using NMP as the template, the nanoscale SAPO-35 samples with a Si content of as low as 5% could be prepared under seed-assisted microwave synthetic conditions. Three-dimensional electron diffraction tomography (3D-EDT) confirmed its typical **LEV**-type framework topology (Supplementary Fig. S9). Owing to the decrease in crystal size and Si content, SAPO-35_NMP_n_0.05 kept 100% methanol conversion for 186 min for MTO reaction, which was much longer than the lifetime of conventional SAPO-35 zeolites (Supplementary Fig. S10). So far, conventional SAPO-35 has not been considered as a good catalytic material for MTO reaction because of its limited lifetime. However, by regulating the isolated SiO_4_ tetrahedra in it, SAPO-35 could be another promising candidate material for MTO reactions. In our previous work, we enumerated >80 000 hypothetical ABC-6 zeolite structures consisting of single 6-rings, double 6-rings and cages similar to those in SAPO-34 and SAPO-35 [28]. Several of these predicted structures have been experimentally realized recently via template design. We anticipate that an increasing number of new ABC-6 zeolites, as well as a large number of hypothetical zeolite structures predicted by other computational approaches [44–46], can be realized in the future, among which candidates with suitable cage structures and heteroatom distribution for desired reactions may be identified as promising new catalysts.

## CONCLUSION

In summary, by using NMP as a new template, the Si content in SAPO-35 zeolites could achieve as low as 5%. More importantly, this new template favors more dispersed distribution of isolated SiO_4_ tetrahedra in the framework than conventional templates, which affects the thermal stability and catalytic lifetime of SAPO-35. Therefore, template design provides a new avenue to the challenging regulation of the isolated SiO_4_ tetrahedra in SAPO zeolites, as well as their chemical and physical properties. Furthermore, our work demonstrates the power of high-throughput computations, which are able to unravel the mysterious distribution of isolated SiO_4_ tetrahedra that is inaccessible to any state-of-the-art characterization technique. To date, millions of hypothetical zeolite framework topologies have been predicted using various computational methods, but their framework compositions and heteroatom distributions have not been investigated because of the expensive computational overheads. With the development of computational algorithms and devices, such as the high-throughput algorithms, distributed computing and GPU-accelerated computing techniques, one can expect that enormous zeolite structures with diverse framework compositions and heteroatom distributions, as well as their template molecules, can be predicted in the near future. In this way, high-throughput computations will provide important theoretical guidance to experimental chemists not only in the synthesis of novel zeolite materials but also in the rational tuning of their properties by heteroatom regulation.

## Supplementary Material

nwac094_Supplemental_File
